# Deciphering the let-7c-5p/RRM2 axis in lung adenocarcinoma: expression, prognosis, and immune landscape implications

**DOI:** 10.3389/fonc.2025.1628429

**Published:** 2025-11-20

**Authors:** Jinlong Liu, Jinying Wu, Yu Han, Lele Li, Xingbo Bian, Xialin Sun, Xuefeng Bian, Xin Sun

**Affiliations:** School of Pharmaceutical Sciences, Jilin Medical University, Jilin, China

**Keywords:** let-7c-5p, RRM2, lung adenocarcinoma, clinical prognosis, immune microenvironment

## Abstract

**Objective:**

Lung adenocarcinoma (LUAD) is a subtype of non-small cell lung cancer with a poor prognosis. Ribonucleotide reductase subunit M2 (RRM2) has been implicated in the progression of various cancers, but its role in LUAD remains underexplored. This study aims to elucidate the expression patterns, clinical significance, and regulatory mechanisms of RRM2 in LUAD.

**Methods:**

We conducted a comprehensive analysis of RRM2 expression using data from the Genomic Data Commons Data Portal and The Cancer Genome Atlas. Survival analysis was performed using the KM plotter tool. The starBase database was utilized to identify miRNAs associated with RRM2. Single-cell RNA sequencing data was analyzed to explore the correlation between RRM2 and immune cell infiltration. *In vitro* experiments were conducted to validate the regulatory role of let-7c-5p on RRM2 in LUAD cell lines.

**Results:**

We found that the expression of RRM2 in LUAD tissues was significantly higher than in normal tissues, and its expression was associated with advanced pathological stage and poor overall survival. Additionally, we identified Let-7c-5p as a potential upstream regulator of RRM2, which is down-regulated in LAUD and negatively correlated with RRM2 expression. *In vitro* experiments showed that overexpression of let-7c-5p reduced RRM2 levels and inhibited LUAD cell proliferation and migration. Moreover, RRM2 expression was positively correlated with the infiltration of certain immune cells, suggesting its role in modulating the tumor immune microenvironment.

**Conclusions:**

Our findings suggest that RRM2 is closely associated with the malignant progression of LUAD and may serve as a potential prognostic biomarker with clinical relevance. Furthermore, the let-7c-5p/RRM2 regulatory axis may play an important role in the development and progression of LUAD, representing a promising therapeutic target that warrants further in-depth investigation.

## Introduction

Lung cancer, a formidable adversary in the global health landscape, is characterized by its escalating incidence and mortality rates, posing a profound threat to both human health and economic stability (1, 2). Non-small cell lung cancer (NSCLC), with lung adenocarcinoma (LUAD) as its predominant subtype, constitutes approximately 85% of all lung cancer cases (3–5). Despite advances in surgery, chemotherapy, radiotherapy, and targeted therapies, the five-year survival rate for LUAD patients remains disappointingly low (6–8). The majority of LUAD patients are diagnosed at advanced stages, often with invasion and metastasis, thereby missing the optimal window for surgical intervention (9, 10). The etiology of LUAD is acknowledged as a complex, multifactorial process, and the biological and clinical heterogeneity of LUAD presents a significant challenge for personalized clinical management. Consequently, the identification of LUAD biomarkers is imperative for enhancing early detection and identifying therapeutic targets.

Recent studies have highlighted the critical role of the ribonucleotide reductase subunit M2 (RRM2) gene in the occurrence and progression of various human cancers, including LUAD. The RRM2 gene encodes the regulatory subunit M2 of ribonucleotide reductase (RNR), an enzyme of paramount importance in cancer treatment. RNR is indispensable for the *de novo* synthesis of deoxyribonucleotides, vital for DNA replication and repair (11, 12). There is increasing evidence that RRM2 may be a promising target for lung cancer treatment (13–16). For example, research by Rahman et al. (17) demonstrated that the regulation of RRM2 induces apoptosis in lung cancer cells through the modulation of Bcl-2 expression. Additionally, low expression levels of RRM2 may be used to assess the response of lung cancer to cisplatin-based chemotherapy (18). Zhou et al. (19) conducted a comprehensive pan-cancer analysis to elucidate the expression patterns, clinical significance, and prognostic value of RRM2 across multiple cancer types, with a particular focus on LUAD. They integrated data from The Cancer Genome Atlas (TCGA), GEO, and other databases to analyze RRM2 expression, clinical pathological features, and survival outcomes. Zhang et al. (20) identified the circ_0039908/miR-let-7c/RRM2 axis as a key regulatory pathway in LUAD, demonstrating that circ_0039908 regulates RRM2 expression through miR-let-7c, thereby affecting LUAD cell proliferation and invasion. However, despite these valuable insights, the specific mechanisms by which RRM2 contributes to LUAD, particularly its interactions with the tumor immune microenvironment, remain underexplored.

Approximately 97% of the human genome is transcribed into non-coding RNA, which can regulate molecular processes at the DNA-RNA-protein level (21–24). This study builds on the foundational work of Zhou et al. (19) and Zhang et al. (20) by focusing on the upstream regulatory mechanisms of RRM2 in LUAD. We predicted the upstream transcription factor let-7c-5p of RRM2 in LUAD cells through bioinformatics analysis and confirmed its abnormal low expression in NSCLC tissues. Let-7 is a widely studied miRNA (25–27), and previous studies have indicated that let-7c-5p is down-regulated in LUAD. High expression of let-7c-5p can significantly inhibit the proliferation of LUAD cells and promote apoptosis (28). Therefore, confirming the relationship between let-7c-5p and RRM2, and understanding how it regulates LUAD development, is crucial for improving the survival rate and quality of life of patients with LUAD.

In this study, we investigated the expression of RRM2 in LUAD and conducted survival analysis. Subsequently, we explored the regulatory mechanisms of RRM2 in LUAD, involving nncRNAs, miRNAs. We elucidated that let-7c-5p can reduce the expression of RRM2, revealing the molecular mechanism by which the let-7c-5p/RRM2 axis regulates LUAD cells. We also examined the biological functions of RRM2 in LUAD. Finally, we established the correlation between RRM2 expression in LUAD and immune cell infiltration, biomarkers, and immune checkpoints. Our findings indicate that the up-regulation of RRM2 mediated by ncRNAs is associated with poor prognosis and tumor immune infiltration in LUAD patients. These observations hold significant implications for basic research and clinical applications and may enhance the precision of immunotherapy for LUAD.

## Materials and methods

### Comparison of the expression differences of RRM2 in LUAD and standard tissues

We employed the Biomarker Exploration of Solid Tumors (BEST) network tool to juxtapose RRM2 expression levels between lung cancer and standard tissues within the GSE68571 and TCGA-LUAD datasets. The gene expression data was standardized by converting them into Z-scores to facilitate comparative analysis (29, 30).

### Analysis of the association of RRM2 and LUAD clinicopathological parameters

We downloaded STAR-counts data and corresponding clinical information for TCGA-LUAD tumors from the TCGA database (https://portal.gdc.cancer.gov). We then extracted data in TPM format and performed normalization using the log2(TPM + 1) transformation. After retaining samples that included both RNA seq data and clinical information, we ultimately selected 516 samples for further analysis. The GTEx data we used is from the V8 version, detailed information can be found on the official GTEx website (https://gtexportal.org/home/datasets). Statistical analysis was conducted using R software, version v4.0.3. Results were considered statistically significant when the *p*-value was less than 0.05 (30).

### Survival analysis

To evaluate the prognostic significance of RRM2 mRNA expression in LUAD, we first assessed its association with overall survival (OS) in patients with lung cancer. Survival analysis was performed using the KM plotter online platform (http://www.kmplot.com/analysis/) (31), The Biomarker Exploration of Solid Tumors (BEST) network tool was used to compare RRM2 expression levels between tumor and normal tissues in the GSE68571 dataset. Gene expression data were standardized by conversion to Z-scores to facilitate cross-sample comparison. In addition, hazard ratios (HRs), log-rank p-values, and 95% confidence intervals (CIs) were calculated.

We then downloaded STAR-counts RNA-seq data and corresponding clinical information for LUAD samples from the TCGA database (https://portal.gdc.cancer.gov). The data were transformed into TPM format and normalized using a log2(TPM + 1) transformation. After filtering to include only samples with both RNA-seq and clinical data, a total of 516 samples were retained for downstream analyses. Consistency validation was further performed using the GEO dataset (GSE68571). Univariate and multivariate Cox proportional hazards regression analyses were conducted to identify independent prognostic factors. Forest plots were generated using the “forestplot” R package to visualize *p*-values, HRs, and 95% CIs for each variable. Based on the multivariate Cox regression results, a prognostic nomogram was constructed using the “rms” package to predict the 5-year overall recurrence probability. All statistical analyses were performed using R software (version 4.0.3), and results with a *p*-value < 0.05 were considered statistically significant.

### Candidate miRNA prediction

Upstream binding miRNAs of RRM2 were predicted by several target gene prediction programs, consisting of PITA, RNA22, miRmap, microT, miRanda, PicTar, and TargetScan. Only the predicted miRNAs that commonly appeared in more than two programs as mentioned above were included for subsequent analyses (32). These predicted miRNAs were regarded as candidate miRNAs of RRM2.

### Correlations between RRM2 and the immune environment

We downloaded STAR-counts data and corresponding clinical information for LUAD tumors from the TCGA database (https://portal.gdc.cancer.gov). Gene expression was converted to TPM and log-transformed (log2[TPM + 1]). Samples with both RNA-seq and clinical data were retained (n = 516) ssGSEA scoring. Immune-cell signatures (published marker gene sets) were scored per sample using GSVA (R) with method = “ssgsea”. Scores represent relative abundance of immune populations.

Group comparison and correlations. Samples were stratified into RRM2-high vs. RRM2-low (median split). Differences in ssGSEA scores between groups were tested by two-sided Wilcoxon rank-sum. Associations between RRM2 expression and immune-cell scores were quantified by Spearman’s ρ. Significance was set at *P* < 0.05 (two-sided). Immune-infiltration patterns were cross-checked using TIMER and TISIDB. TIMER’s SCNA module was used to compare infiltration across RRM2 copy-number states (deep deletion, arm-level deletion, diploid/normal, arm-level gain, high amplification), using TIMER’s default statistics (33).

### Single-cell RNA-seq

The data used in the above research all come from the files in the **Supplementary File** of the GEO database (https://www.ncbi.nlm.nih.gov/geo/). The Seurat package was utilized to generate objects and filter out cells of poor quality, while also carrying out a standard data preprocessing procedure. The number of genes, the number of cells, and the percentage of mitochondrial content were calculated. The filtering criteria were genes detected in fewer than 5 cells and cells with fewer than 200 detected genes.

We retain genes detected in at least 12346 cells and filter out cells with fewer than 200 detected genes, as well as cells with a high mitochondrial content (>5%). After discarding low-quality cells, we retain 2000 cells for downstream analysis. To normalize each cell, we scale the UMI counts using scale factor = 10,000. After logarithmically transforming the data, we use the ScaleData function (v4.1.0) in Seurat.

We apply the corrected normalized data to standard analysis, extracting the top 2000 variable genes for Principal Component Analysis (PCA). For the visualization and clustering of UMAP (or TSNE), we retain the top 20 principal components. Cell clustering is performed using the FindClusters function (resolution = 0.8) implemented in the Seurat R package (34).

### Cell culture, antibodies, siRNA and plasmids

The A549 and H460 cells were incubated at 37°C in a 5% CO_2_ atmosphere. The primary antibodies for RRM2 polyclonal antibody were purchased from Cell Signaling Technology (Danvers, MA, United States). The β-actin and secondary antibodies were purchased from Protein Technology Group, Inc. (Wuhan, China). The siRNA of RRM2 were purchased from jtsbio Biotech (jtsbio Biotech Co.,Ltd, Wuhan, China). jtsbio Biotech designed and established the let-7c-5p overexpression plasmid (jtsbio Biotech Co.,Ltd, Wuhan, China) (**Supplementary Table 1**). Plasmid and siRNA transfection was performed with Lipofectamine^®^ 3000 following the manufacturer’s instructions.

### CCK8 assay

Cells were seeded at a density of 5 × 10^3^ cells/well. Add 10 µl of CCK8 solution at 24, 48, and 72 hours, and measure the absorbance at 450 nm after 2 hours.

### Transwell assay

Cells were seeded at a density of 4×10^4^ cells in 24-well transwell inserts. After 24 hours, they were fixed with 4% paraformaldehyde for 10 minutes, stained with crystal violet solution for 12 minutes, and images were collected.

### Quantitative real-time PCR

Cells were collected, and total RNA was extracted using Trizol reagent (Invitro-gen Inc., Carlsbad, CA) according to the manufacturer’s protocol. The expression levels of let-7c-5p and RRM2 mRNA were normalized to the GAPDH (**Supplementary Table 2**). The relative fold changes in target gene expression between the control group and the experimental group were calculated using the 2^−ΔΔCT^ method.

### Western blot assays

Homogenize cells in RIPA lysis buffer. Determine protein concentration using a BCA assay kit, boil the mixture at 98 °C in a Dual-Color protein loading buffer, separate equal amounts of protein by SDS-PAGE, and transfer to a PVDF membrane. Subsequently, incubate the membrane with primary and secondary antibodies. Perform enhanced chemiluminescence to visualize the protein bands. Finally, apply ImageJ for quantitative analysis.

### Statistical analysis

All results are expressed as mean ± SD. Comparisons among groups were conducted using one-way ANOVA and Tukey’s multiple comparison tests. A *p*-value of less than 0.05 was considered statistically significant. Data analysis was performed using the BEST, TIMER, and TISIDB platforms, which automatically applied the Benjamini-Hochberg method for FDR correction in multiple hypothesis testing and reported effect sizes (such as correlation coefficients and normalized enrichment scores) in correlation and enrichment analyses. Analyses were performed in R 4.0.3 using GSVA and base/ggplot2 functions for statistics and plotting.

## Results

### RRM2 overexpression drives tumor progression and predicts poor prognosis in LUAD

**Figure 1:**
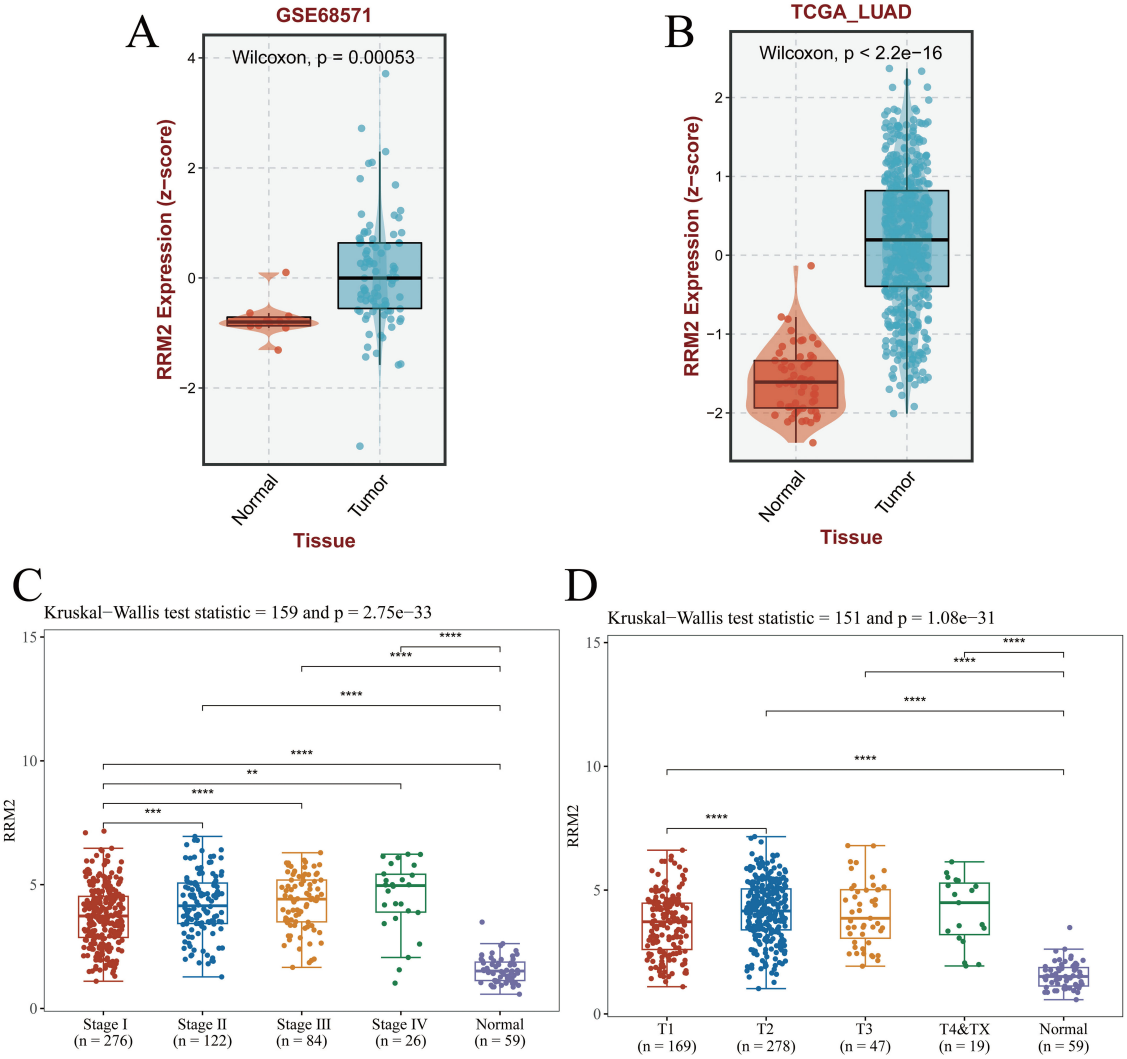
RRM2 expression in LUAD and its clinical implications. **(A, B)** The GEO dataset and TCGA-LUAD dataset download from the BEST database showed that RRM2 was highly expressed in LUAD. **(C)** Pathologic stage. **(D)** Pathologic T stage. (*p<0.05; **p<0.01; ***p<0.001, ns, no significance).

Transcriptomic profiling of LUAD tissues from the GSE68571 and TCGA-LUAD cohorts demonstrates significant up-regulation of RRM2 in tumors compared to normal tissues (Wilcoxon test, *p*=0.00053 and *p*<2.2×10^-16^, respectively; **Figures 1A, B**). RRM2 expression levels were found to escalate with advancing tumor stage (Kruskal-Wallis test, *p*=2.273×10^-33^), and correlated strongly with TNM-T classification (*p*=1.08×10^-31^), suggesting its role in metastatic progression (**Figures 1C, D**). Collectively, these findings indicate that RRM2 may contribute to the malignant progression of LUAD and could represent a potential therapeutic target. Its stage-dependent overexpression and significant association with patient survival highlight the potential clinical utility of RRM2 for risk stratification and precision oncology applications.

### RRM2 overexpression predicts poor prognosis and serves as an independent prognostic factor in LUAD

**Figure 2:**
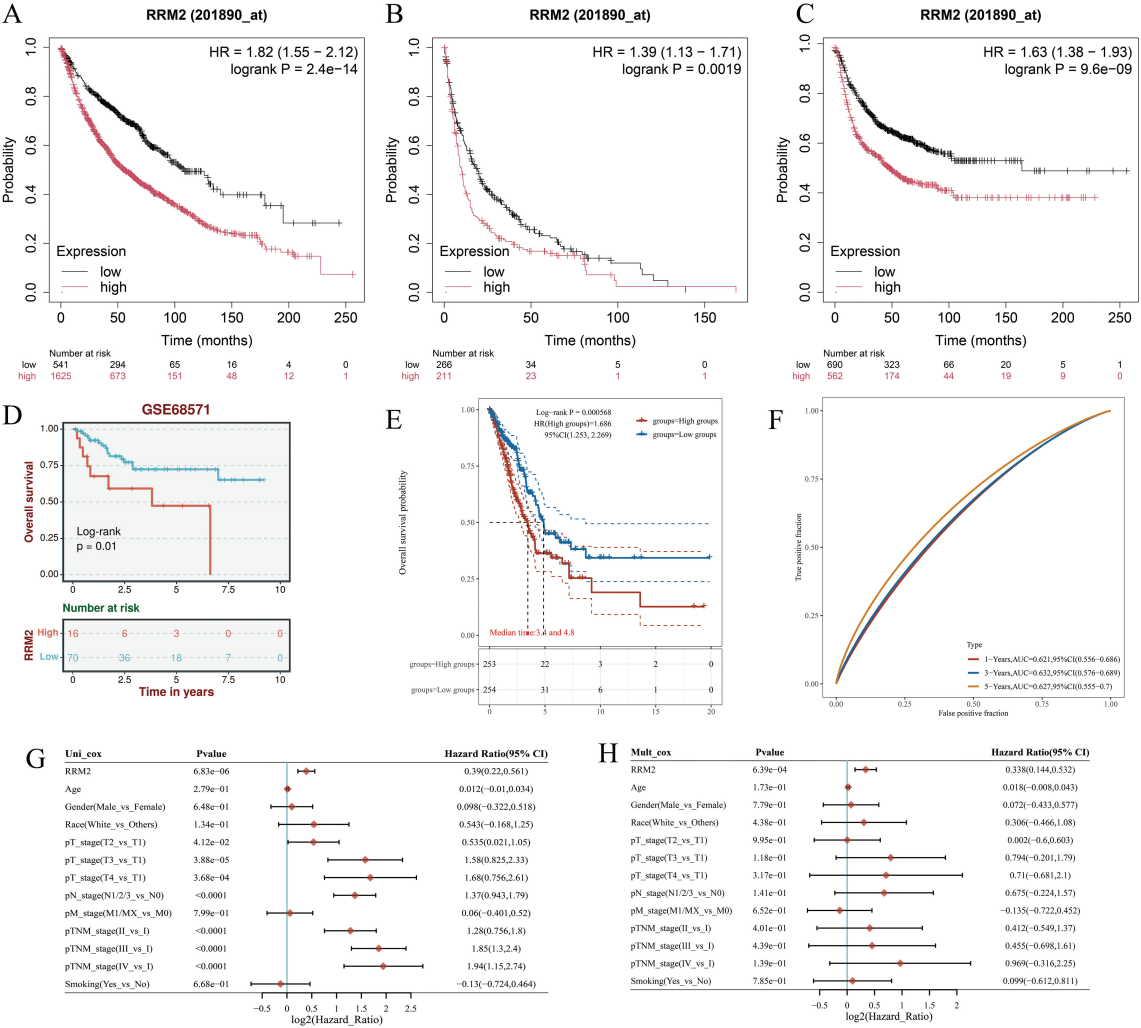
Prognostic value of RRM2 in LUAD. **(A)** Overall survival analysis of RRM2 mRNA high and low expression in LUAD. **(B)** Pps analysis of RRM2 mRNA high and low expression in LUAD. **(C)** FP analysis of RRM2 mRNA high and low expression in LUAD. **(D)** Survival curves of high and low RRM2 expression in the GsE68571. **(E)** The KM survival curve of the gene in TCGA data. **(F)** Time-dependent ROC curve. **(G)** Univariate and **(H)** multivariate COX regression analysis of OS correlation in LUAD. Fp, First Progression; OS, Overall Survival; PpS, Post Progression Survival.

Kaplan–Meier survival analyses consistently demonstrated that patients with high RRM2 expression had significantly worse OS compared with those with low expression across multiple datasets. In the TCGA-LUAD cohort, elevated RRM2 levels were strongly associated with reduced survival probability (HR = 1.82, 95% CI: 1.55–2.12, *p* = 2.4 × 10^-14^; **Figure 2A**), which was further confirmed in two additional LUAD datasets (HR = 1.39, 95% CI: 1.13–1.71, *p* = 0.0019; HR = 1.63, 95% CI: 1.38–1.93, *p* = 9.6 × 10^-9^; **Figures 2B, C**). The external validation cohort (GSE68571) also supported these findings, showing significantly shorter OS in the high RRM2 group (log-rank *p* < 0.01; **Figure 2D**).Time-dependent ROC curve analysis yielded AUC values of 0.621 (1-year), 0.597 (3-year), and 0.586 (5-year), indicating limited but consistent prognostic discrimination across time points (**Figures 2E, F**).Univariate Cox regression identified RRM2, TNM stage, and smoking status as significant predictors of OS (**Figure 2G**). Importantly, multivariate Cox analysis confirmed RRM2 as an independent prognostic factor for LUAD (HR = 1.30, 95% CI: 1.14–1.53, *p* < 0.01), after adjusting for age, sex, race, tumor stage, and smoking status (**Figure 2H**). Collectively, these findings demonstrate that RRM2 overexpression is significantly associated with unfavorable clinical outcomes and may serve as an independent prognostic biomarker in LUAD, providing potential value for risk stratification and precision oncology applications.

### Prediction and analysis of upstream miRNAs of RRM2

**Figure 3:**
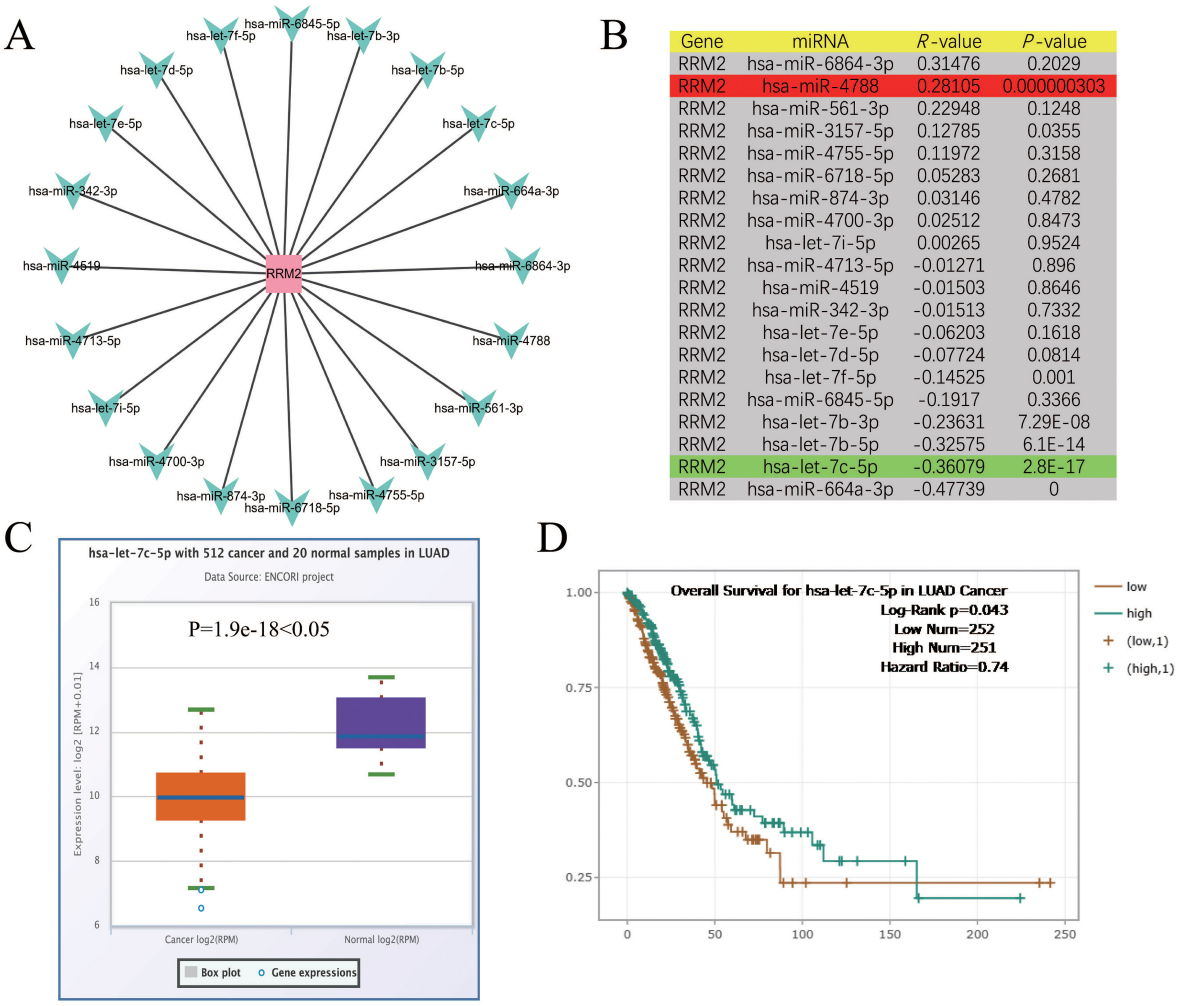
Identification of let-7c-5p as a potential upstream regulatory miRNA of RRM2 in LUAD. **(A)** The miRNA-RRM2 prediction network produced by Cytoscape. **(B)** The correlation between the predicted expression of some candidate miRNAs and RRM2 in LUAD analyzed by starBase. **(C, D)** The expression and prognostic value of let-7c-5p in LUAD were detected by starBase and Kaplan Meier plotter.

The pivotal role of ncRNAs, notably miRNAs in modulating gene expression is well-established. In LUAD, RRM2 has been associated with tumorigenesis, and its expression is hypothesized to be under miRNA-mediated control. To probe this hypothesis, an exhaustive analysis was undertaken to uncover miRNAs capable of binding and modulating RRM2 expression. Utilizing Cytoscape software, a network was delineated to graphically represent the interactions between RRM2 and a spectrum of miRNAs. This network schematizes the putative regulatory relationships between RRM2 and 20 distinct miRNAs, encompassing let-7f-5p, miR-6845-5p, let-7b-3p, among others. The connections between RRM2 and these miRNAs imply potential regulatory influences they may exert on RRM2 expression (**Figure 3A**). This visualization serves as a foundation for further investigation into the intricate interplay between RRM2 and miRNAs in the context of LUAD.

To delve deeper into the interplay between RRM2 and the aforementioned miRNAs, an expression correlation analysis was meticulously conducted. The findings, tabulated and presented with correlation coefficients (R-values) and their corresponding statistical significance (*P*-values), delineate the relationship between each miRNA-RRM2 pair. Notably, RRM2 exhibited significant inverse correlations with let-7f-5p (R = -0.14525, *P* = 0.001), let-7b-3p (R = -0.23631, *P* = 7.29E-08), and let-7b-5p (R = -0.32575, *P* = 6.1E-14), suggesting that these miRNAs may function as tumor suppressors by negatively regulating RRM2 expression. In contrast, a positive correlation was between RRM2 and miR-4788 (R = 0.28105, *P* = 0.000000303), implying a potential oncogenic role in enhancing RRM2 expression (**Figure 3B**). Focusing on let-7c-5p, a miRNA of particular interest due to its significant negative correlation with RRM2, we compared its expression levels between 512 LUAD cancer samples and 20 standard samples using box plots. The analysis revealed a substantial down-regulation of let-7c-5p in cancer samples (*P* = 1.9e-18), hinting that its diminished expression might contribute to the up-regulation of RRM2 and potentially foster tumorigenesis (**Figure 3C**). The prediction significance of let-7c-5p in LUAD was further evaluated using KM survival plots. The analysis indicated that patients with elevated let-7c-5p expression levels exhibited improved OS rates (Log-Rank *p* = 0.043, Hazard Ratio = 0.74), suggesting that let-7c-5p could serve as a potential prediction biomarker and therapeutic target for LUAD (**Figure 3D**). These results underscore the intricate regulatory network involving RRM2 and miRNAs in LUAD and highlight the potential of miRNAs as therapeutic agents.

### let-7c-5p regulates RRM2 to inhibit the progression of LUAD

**Figure 4:**
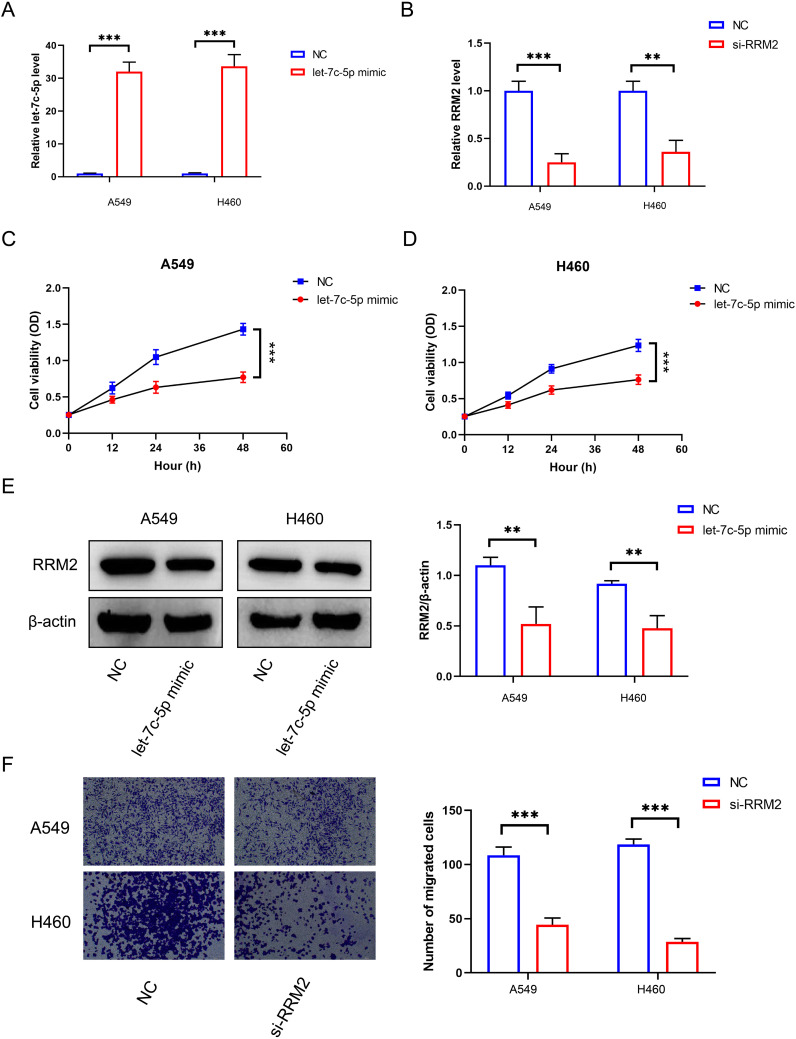
The effect of let-7c-5p regulation on RRM2 in LUAD. **(A)** The let-7c-5p expression in A549 and H460 cells after let-7c-5p mimic/control transfection was determined by RT-qPCR. **(B)** RRM2 siRNA transfection efficiency was detected by qRT-PCR. **(C, D)** Proliferation of A549 and H460 cells after let-7c-5p transfection determined by the CCK8 assay. **(E)** Let-7c-5p mimic could decrease the expression of RRM2. **(F)** Blocking RRM2 expression could decrease the metastasis number of A549 and H460 cells (*p < 0.05, **p < 0.01, ***p < 0.001).

To verify the role of let-7c-5p in the development of NSCLC, we measured the expression of let-7c-5p in NSCLC cell lines H273, H23, A549, and H460, as well as in normal lung epithelial cells 16HBE, using RT-qPCR. The results showed that the expression of let-7c-5p was lower in all NSCLC cell lines compared to 16HBE cells (**Supplementary Figure S1**). Among them, the A549 and H460 cells showing the lowest expression of let-7c-5p were transfected with let-7c-5p mimic or mimic control for subsequent use (**Figure 4A**). RT-qPCR assay presented the transfection effectiveness of RRM2 siRNA in A549 and H460 cells (**Figure 4B**). The results of the CCK8 analysis indicate that the number of proliferating A549 and H460 cells decreases following the overexpression of let-7c-5p (**Figures 4C, D**). From a molecular perspective, it was found that the let-7c-5p mimic reduces the level of RRM2 (**Figure 4E**). The transwell experiment demonstrated that the absence of RRM2 significantly inhibited the cell migration and metastasis capabilities (**Figure 4F**). The above results indicate that let-7c-5p regulates RRM2, which inhibits the proliferation and migratory capacity of LUAD cells *in vitro*.

### Bulk immune infiltration associated with RRM2 expression

**Figure 5:**
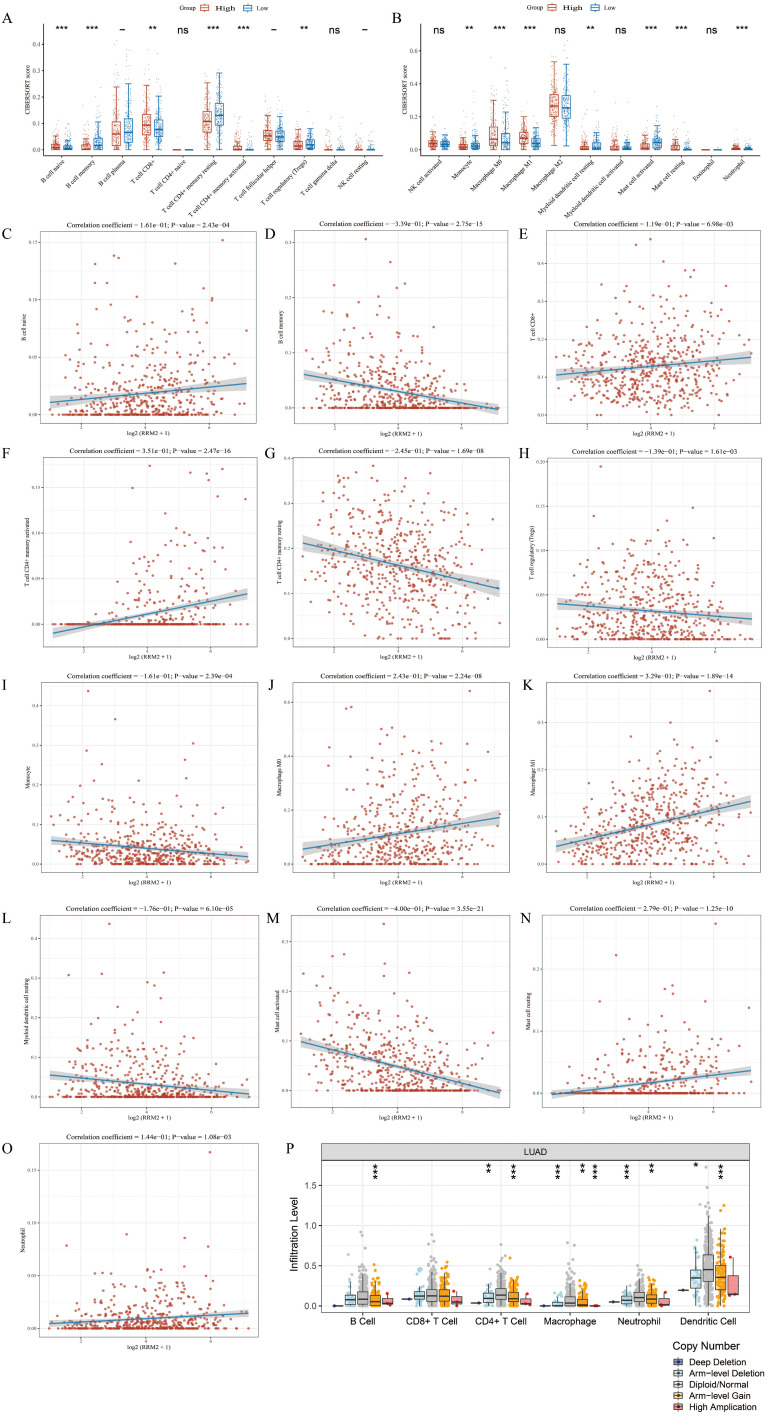
Association between RRM2 and immune cell infiltration in LUAD. **(A, B)** According to different expression levels of RRM2, the infiltration levels of immune cells were analyzed in groups. **(C-O)** RRM2 is correlated with immune infiltration in LUAD. **(P)** The relationship between the altered somatic copy number of RRM2 gene and infiltrating immune cells in LUAD (*p < 0.05; **p < 0.01; ***p < 0.001, ns, no significance).

Utilizing the ssGSEA methodology, we quantified the associations between RRM2 expression and the levels of immune cell infiltration in LUAD (**Figures 5A, B**). Our findings revealed a positive correlation between RRM2 expression and the infiltration of Natural Killer (NK) CD56bright cells, TH2 cells, TFH, T effector memory cells (Tem), and Macrophages, while a negative correlation was with Th17 cells, dendritic Cells (DCs), neutrophils, cytotoxic cells, regulatory T cells (Tregs), T memory cells (Tcm), pDCs, CD8^+^ T cells, and B cells. To further elucidate these relationships, scatter plots were employed to illustrate the correlation between RRM2 expression and various immune cell populations in LUAD (**Figures 5C–O**). These plots include: B cells (**Figure 5C**), CD4^+^ T cells (**Figure 5D**), CD8^+^ T cells (**Figure 5E**), Macrophages (M0) (**Figure 5F**), Macrophages (M1) (**Figure 5G**), DCs (**Figure 5H**), Neutrophils (**Figure 5I**), Cytotoxic Cells (**Figure 5J**), Tregs (**Figure 5K**), Tcm (**Figure 5L**), Tem (**Figure 5M**), NK cells (**Figure 5N**), Monocytes (**Figure 5O**). Box plots (**Figure 5P**) were used to display the infiltration levels of different immune cells in LUAD, categorized by copy number alterations. These analyses indicate that the expression level of RRM2 is closely associated with the immune cell infiltration patterns in LUAD. The correlation coefficients and *P*-values provide a quantitative measure of the strength and statistical significance of these associations. Our data imply that elevated RRM2 expression may be associated with a subdued anti-tumor immune response. The use of TIMER and TISIDB databases further corroborates the connection between RRM2 expression and immune cell infiltration, potentially influencing LUAD prognosis and treatment responses. These findings highlight the complex interplay between RRM2 expression and immune cell dynamics in LUAD and may have implications for developing immunotherapeutic strategies.

### Single-cell mapping of RRM2 across immune subsets

**Figure 6:**
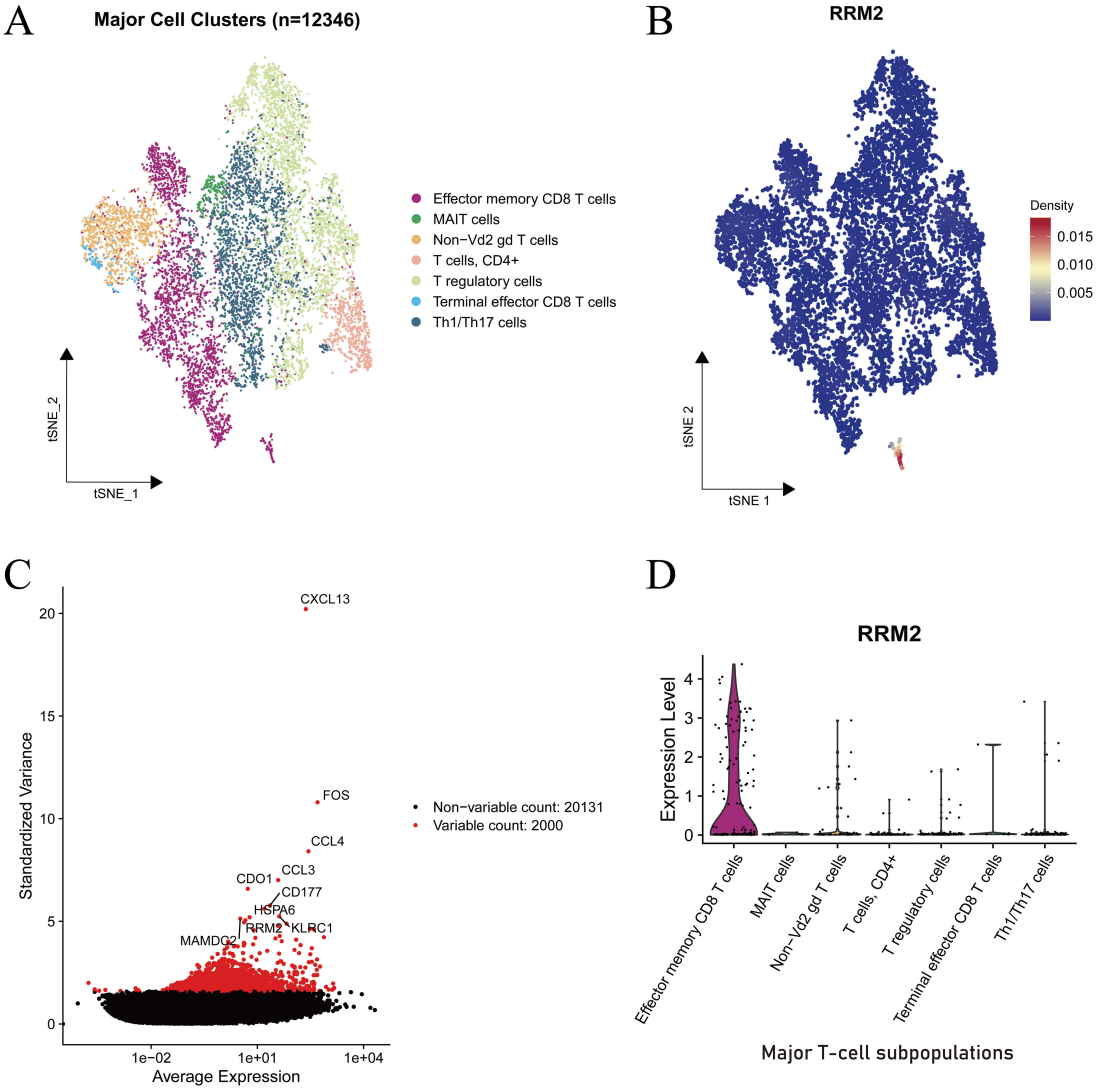
Single-cell transcriptomic characterization of RRM2 expression in immune cell subsets of LUAD. **(A)** Cell type lineage mapping. **(B)** Density map of RRM2 expression projected onto the t-SNE embedding. **(C)** Mean variance relationship of gene expression levels. **(D)** Violin plot depicting RRM2 expression across major T-cell subsets.

To delineate the immune landscape of LUAD, single-cell transcriptomic data comprising 12,346 cells were analyzed using t-distributed stochastic neighbor embedding (t-SNE). This dimensionality reduction approach revealed seven transcriptionally distinct T-cell clusters, including effector memory CD8 T cells, MAIT cells, Non-Vd2 γδ T cells, CD4^+^ T cells, T regulatory cells, Terminal effector CD8 T cells, and Th1/Th17 cells (**Figure 6A**). The clear separation of these clusters in the two-dimensional embedding underscores the intrinsic transcriptional heterogeneity and functional specialization of the T-cell compartment in LUAD.

To identify genes contributing to cellular heterogeneity, the relationship between standardized variance and average expression was examined (**Figure 6B**). Among analyzed, 2,000 were classified as highly variable (highlighted in red). Notably, RRM2 emerged among the top variable genes, alongside immune activation associated genes such as FOS, CCL3, CD177, and HSPA6, indicating its potential involvement in immune cell proliferation or activation processes.

The spatial distribution map of RRM2 expression across the t-SNE projection (**Figure 6C**) demonstrated a strikingly localized pattern, with the highest density observed within a small subset of cells positioned at the lower region of the embedding. This subpopulation corresponded to the effector memory CD8 T-cell cluster, while most other immune subtypes exhibited minimal RRM2 expression. The density gradient further confirmed a concentrated enrichment of RRM2-positive cells, suggesting that RRM2 expression is functionally confined to a proliferative or metabolically active subset.

Violin plot analysis (**Figure 6D**) corroborated these findings, showing that RRM2 expression was markedly elevated in effector memory CD8 T cells, whereas it remained low or undetectable in MAIT cells, T regulatory cells, Th1/Th17 cells, and other subsets. Given the established role of RRM2 in deoxyribonucleotide synthesis and DNA repair, its preferential expression in effector memory CD8 T cells likely supports enhanced proliferative potential and metabolic demand during immune activation.

Collectively, these data identify RRM2 as a proliferation-associated marker preferentially expressed in activated cytotoxic T cells, implicating its involvement in sustaining immune effector responses within the LUAD tumor microenvironment (TME).

## Discussion

The present study provides a comprehensive analysis of the role of RRM2 in LUAD and its interplay with miRNAs and the tumor immune microenvironment. Our findings highlight the multifaceted role of RRM2 in LUAD pathogenesis, prognosis, and immune regulation. The prediction and analytical exploration of upstream miRNAs targeting RRM2 has shedlight on potential regulatory mechanisms involving ncRNAs, which are recognized for their role in modulating gene expression in cancer. Notably, miRNAs such as let-7f-5p, let-7b-3p, and let-7b-5p exhibited significant negative correlations with RRM2, implying their roles as tumor suppressors by down-regulating RRM2. In contrast, the positive correlation between RRM2 and miR-4788 suggests a role in enhancing RRM2 expression, potentially contributing to oncogenic processes. Our findings indicate that let-7c-5p can repress RRM2 expression, and the inhibition of RRM2 can curtail the metastatic potential of LUAD cells. This observation is congruent with the outcomes of our comprehensive analysis, highlighting the relevance of these regulatory interactions in LUAD pathobiology.

Our integrated multi-omics analysis revealed that RRM2 is significantly up-regulated in LUAD tissues compared to normal counterparts, with a progressive increase in expression associated with advanced tumor stages and TNM classification. This up-regulation of RRM2 is consistent with its known role in promoting cell proliferation and DNA synthesis, which are critical for tumor growth and metastasis. In this study, elevated RRM2 expression was found to be significantly associated with poorer OS in patients with LUAD, underscoring its potential prognostic relevance. The ROC analysis yielded an AUC of 0.62, suggesting limited discriminative ability; therefore, RRM2 alone may not serve as a reliable standalone diagnostic biomarker. The Kaplan–Meier survival analysis, using the median expression value as the cutoff, further supported its association with unfavorable prognosis. Taken together, these findings indicate that RRM2 holds promise as a prognostic biomarker, although its diagnostic utility appears limited and requires further validation in larger, independent cohorts.

**Figure 7:**
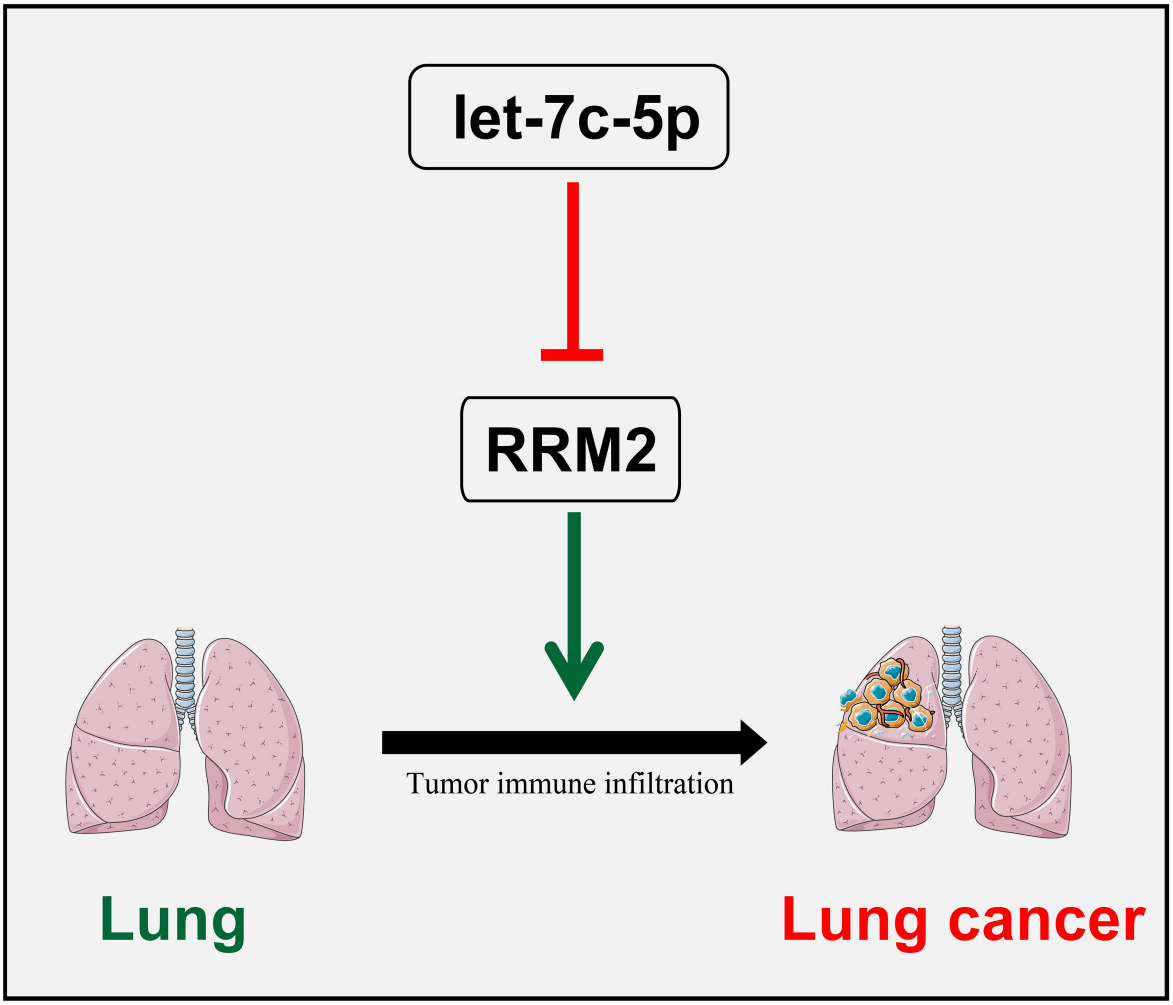
The schematic diagram of let-7c-5p/RRM2 in LUAD.

The identification of miRNAs that regulate RRM2 expression provides insights into the post-transcriptional mechanisms underlying LUAD progression. Our analysis revealed several miRNAs, including let-7f-5p, let-7b-3p, and let-7b-5p, that exhibit significant inverse correlations with RRM2 expression. These miRNAs are likely to function as tumor suppressors by negatively regulating RRM2. In particular, let-7c-5p, which showed a strong negative correlation with RRM2, was significantly down-regulated in LUAD samples. The prognostic significance of let-7c-5p, as demonstrated by improved OS in patients with higher expression levels, highlights its potential as a therapeutic target and biomarker. The restoration of let-7c-5p expression could potentially inhibit RRM2, thereby suppressing tumor progression in LUAD (**Figure 7**).

Our experimental validation further elucidated the role of let-7c-5p in the progression of LUAD. We observed that, compared with normal lung epithelial cells (16 HBE), A549 and H460 cells exhibited the lowest levels of let-7c-5p expression and were subsequently transfected with let-7c-5p mimics or controls for functional studies. The overexpression of let-7c-5p in these cells significantly reduced RRM2 expression, as confirmed by western blot analyses. The decrease in RRM2 levels was associated with reduced cell migration capabilities, as demonstrated by transwell assays. These results directly prove that let-7c-5p regulates RRM2 expression, thereby inhibiting the proliferation and migratory capacity of LUAD cells *in vitro*. This regulatory mechanism highlights the tumor-suppressive role of let-7c-5p and underscores its potential as a therapeutic target for LUAD.

The correlation analysis between RRM2 expression and immune cell infiltration revealed a complex and multifaceted interaction within the tumor immune microenvironment of LUAD. Elevated RRM2 expression was positively correlated with the infiltration of specific immune subsets, such as NK CD56^+^bright cells and Th2 cells, while showing negative correlations with Th17 cells and DCs. This bidirectional association suggests that RRM2 may contribute to shaping the immune landscape of LUAD, potentially through influencing immune cell recruitment and activation dynamics.

Given the pivotal role of the TME in determining immunotherapy efficacy, RRM2-associated proliferative activity and variations in myeloid and lymphoid cell composition may confound observed immune response signals (35, 36). Therefore, future studies should integrate proliferation indices, cytokine signaling pathways, and myeloid lineage markers to better distinguish association from causation, and to evaluate whether RRM2 adds prognostic value to composite models of immunotherapy response (37, 38).

At the single-cell level, transcriptomic profiling uncovered marked heterogeneity among T-cell subpopulations, identifying RRM2 as a gene with distinctive expression patterns across immune lineages. Notably, RRM2 was selectively enriched in effector memory CD8^+^ T cells, consistent with its canonical role in deoxyribonucleotide synthesis and DNA replication, which are essential for rapid clonal expansion and sustained cytotoxic activity. In contrast, RRM2 expression was minimal in Tregs and exhausted CD8^+^ T-cell subsets, reinforcing its association with a metabolically active and cytotoxic immune phenotype rather than with immunosuppressive or dysfunctional states.

Collectively, these findings position RRM2 as a proliferation-associated and functionally relevant marker of activated cytotoxic T cells, potentially contributing to antitumor immune responses in LUAD. The dual correlations observed across innate and adaptive immune compartments further imply that RRM2 may act as a molecular modulator linking metabolic programming to immune functionality. Future mechanistic studies are warranted to elucidate whether RRM2 directly governs T-cell activation, persistence, or effector function, which could inform the development of novel immunotherapeutic strategies targeting LUAD.

## Conclusion

In summary, our study provides strong evidence for the critical role of RRM2 in LUAD, highlighting its high expression, its association with poor prognosis, and the complex regulatory mechanisms involving miRNAs. The let-7c-5p/RRM2 axis holds promise as a novel therapeutic target for LUAD. Moreover, RRM2-related genetic alterations and the immune microenvironment underscore its multifaceted role in LUAD. These findings warrant further investigation into the mechanisms of RRM2 in LUAD and its potential as a therapeutic target and prognostic biomarker, paving the way for new frontiers in personalized medicine.

## Data Availability

The original contributions presented in the study are included in the article/**Supplementary Material**. Further inquiries can be directed to the corresponding authors.

## Glossary

AUC: The Area Under the Curve
CCK8: Cell Counting Kit-8
BP: Biological processes
BEST: Biomarker Exploration of Solid Tumors
CC: Cellular components
CI: Confidence intervals
DSS: Disease Specific Survival
GSEA: Gene Set Enrichment Analysis
GO: Gene Ontology
HR: Hazard ratios
LUAD: Lung adenocarcinoma
KM: Kaplan-Meier
KEGG: Kyoto Encyclopedia of Genes and Genomes
MF: Molecular functions
OS: Overall survival
PFS: Progression Free Survival
RFS: Recurrence Free Survival
ssGSEA: Single-sample Gene Set Enrichment Analysis
TCGA: The cancer Genome Atlas
TIMER: The Tumor Immune Estimation Resource
DCs: Dendritic Cells
Tregs: Regulatory T cells
Tcm: T memory cells
Tem: T effector memory cells
NK: Natural Killer cells
