# Supplementary material for: Deciphering the let-7c-5p/RRM2 axis in lung adenocarcinoma: expression, prognosis, and immune landscape implications
Source: Front Oncol. 2025 Nov 20;15:1628429. doi: 10.3389/fonc.2025.1628429 (PMC12675248; doi:10.3389/fonc.2025.1628429)
Supplement: Supplementary file 1 [file DataSheet1.zip › Supplementary Materials/GEO Independent Verification - Raw Data/cal_plotGSE68571.pdf]

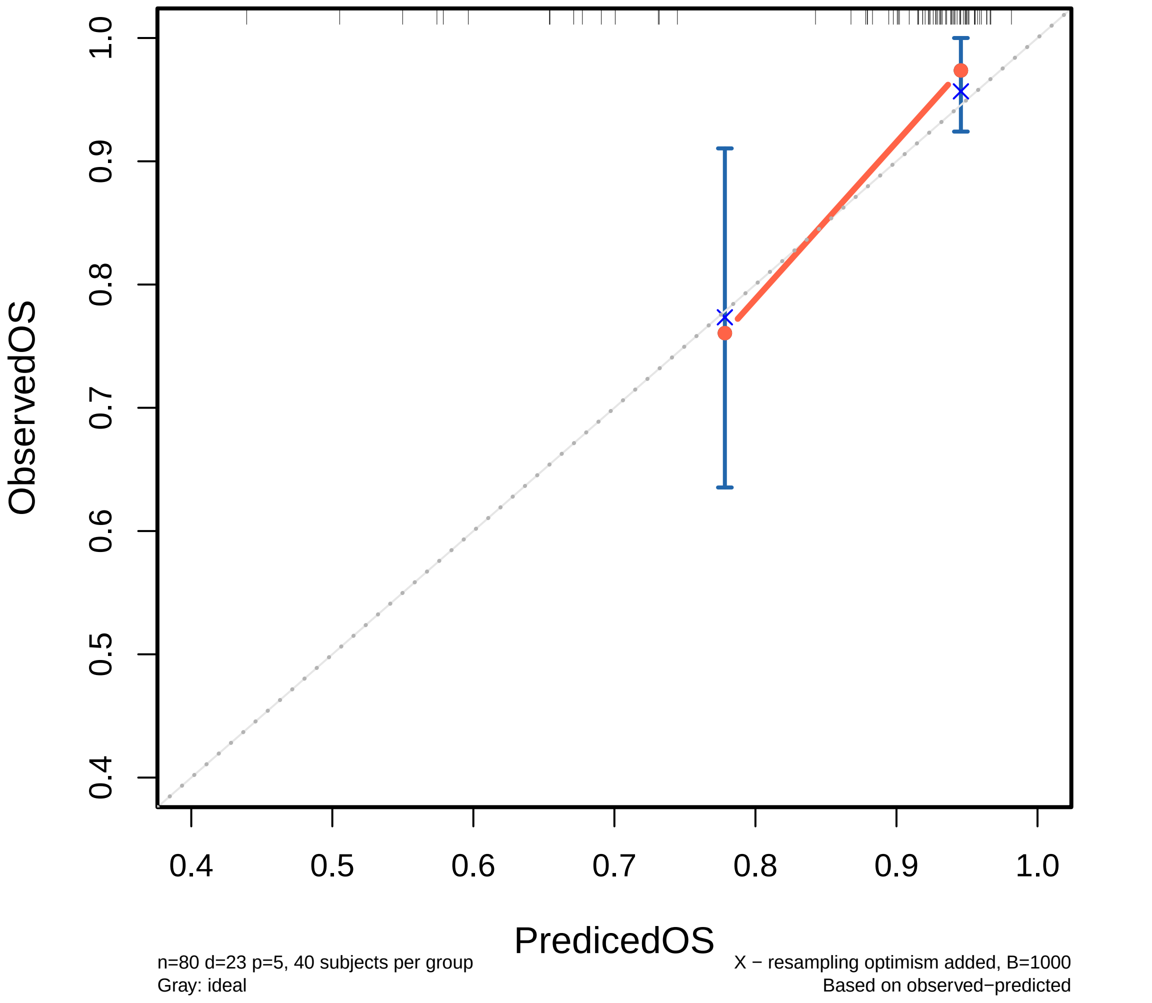

1.0
0.9
0.8
0.7
0.6
0.5
0.4
ObservedOS
0.4
0.5
0.6
0.7
0.8
0.9
1.0
PredicedOS
n=80 d=23 p=5, 40 subjects per group
Gray: ideal
X − resampling optimism added, B=1000
Based on observed−predicted
