# Supplementary figures and images for: Deciphering the let-7c-5p/RRM2 axis in lung adenocarcinoma: expression, prognosis, and immune landscape implications
Source: Front Oncol. 2025 Nov 20;15:1628429. doi: 10.3389/fonc.2025.1628429 (PMC12675248; doi:10.3389/fonc.2025.1628429)

C-index : 0.613 (0.561–0.665) p < 0.001

Points

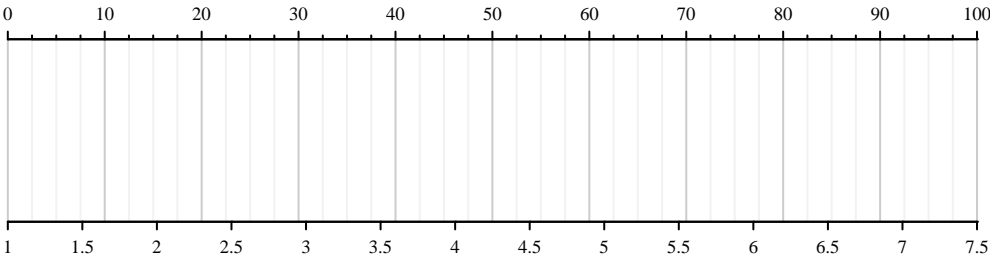

RRM2

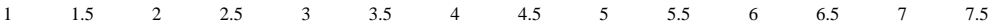

Total Points

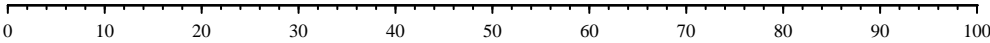

Linear Predictor

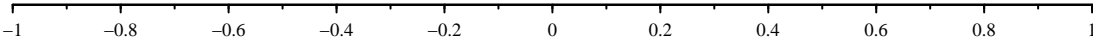

surv1

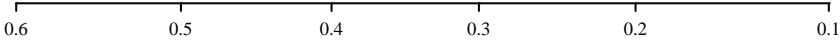

surv3

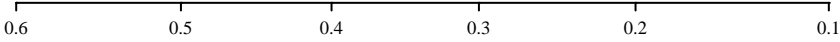

surv5

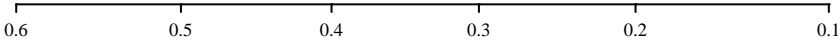

Supplement: Supplementary file 1 [file DataSheet1.zip › Supplementary Materials/Standard curve/Nomogram.pdf]

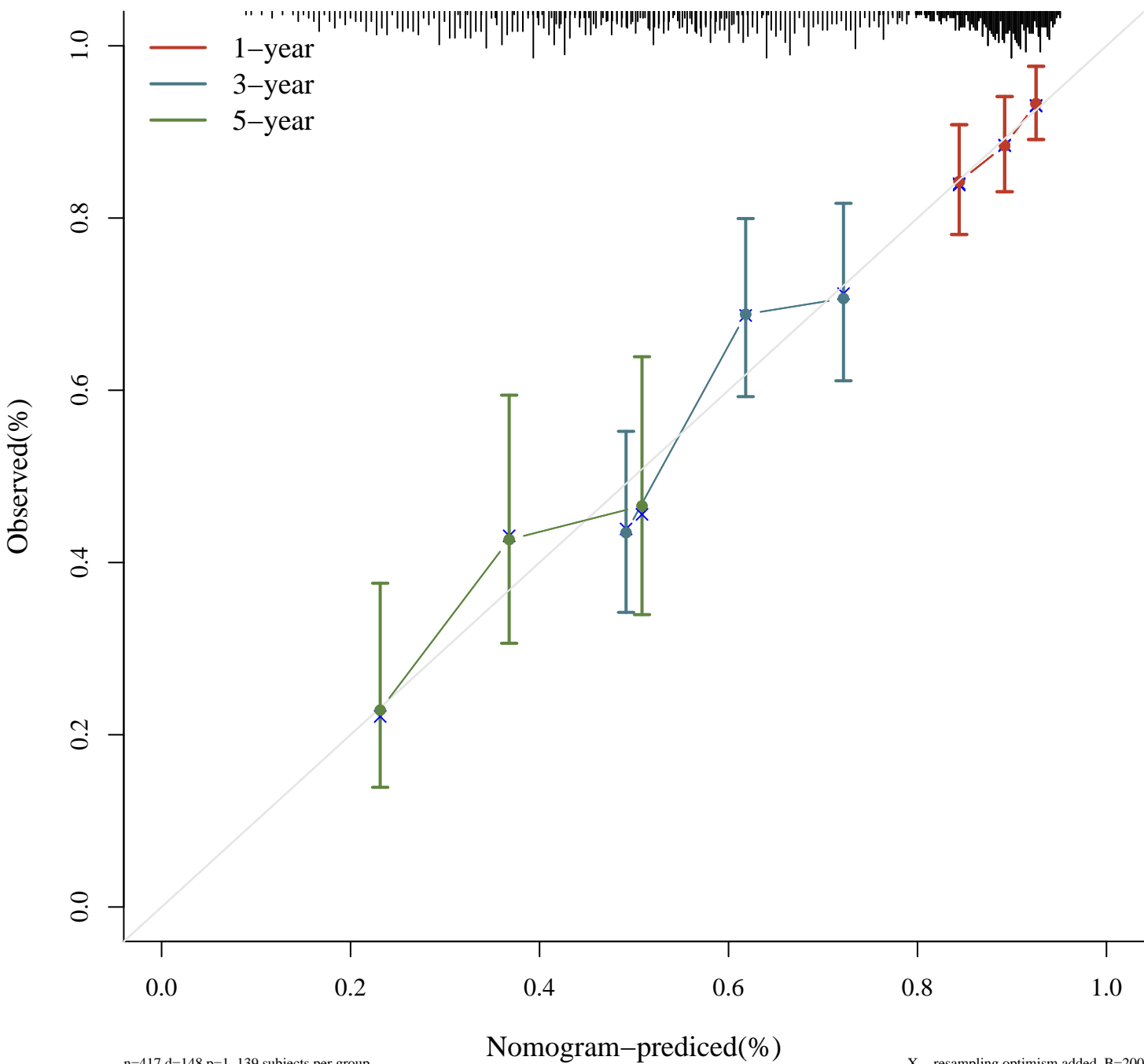

Supplement: Supplementary file 1 [file DataSheet1.zip › Supplementary Materials/Standard curve/Standard curve.pdf]

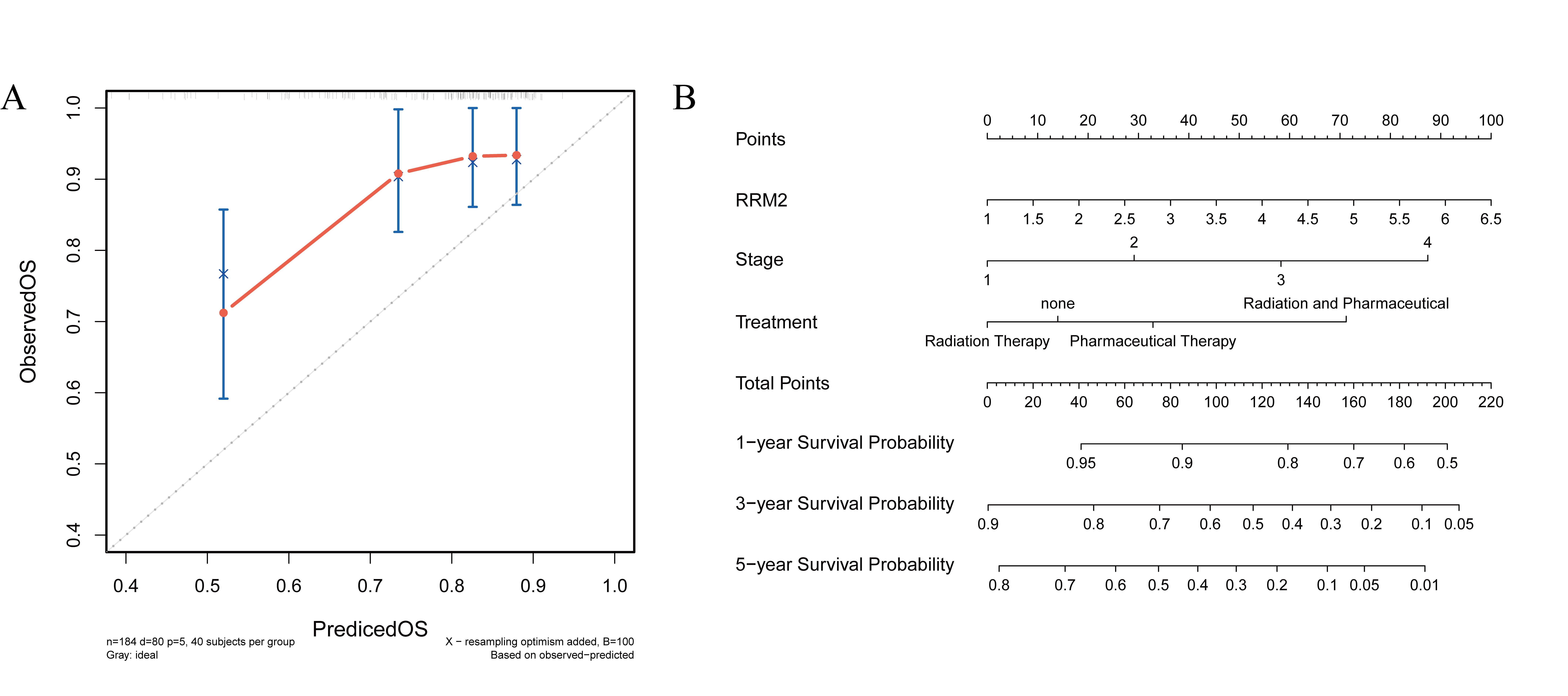

Supplement: Supplementary file 1 [file DataSheet1.zip › Supplementary Materials/Standard Curve and Nomogram/Standard Curve and Nomogram.png]
