# Supplementary material for: Deciphering the let-7c-5p/RRM2 axis in lung adenocarcinoma: expression, prognosis, and immune landscape implications
Source: Front Oncol. 2025 Nov 20;15:1628429. doi: 10.3389/fonc.2025.1628429 (PMC12675248; doi:10.3389/fonc.2025.1628429)
Supplement: Supplementary file 2 [file Presentation1.ppt]

## Slide 1
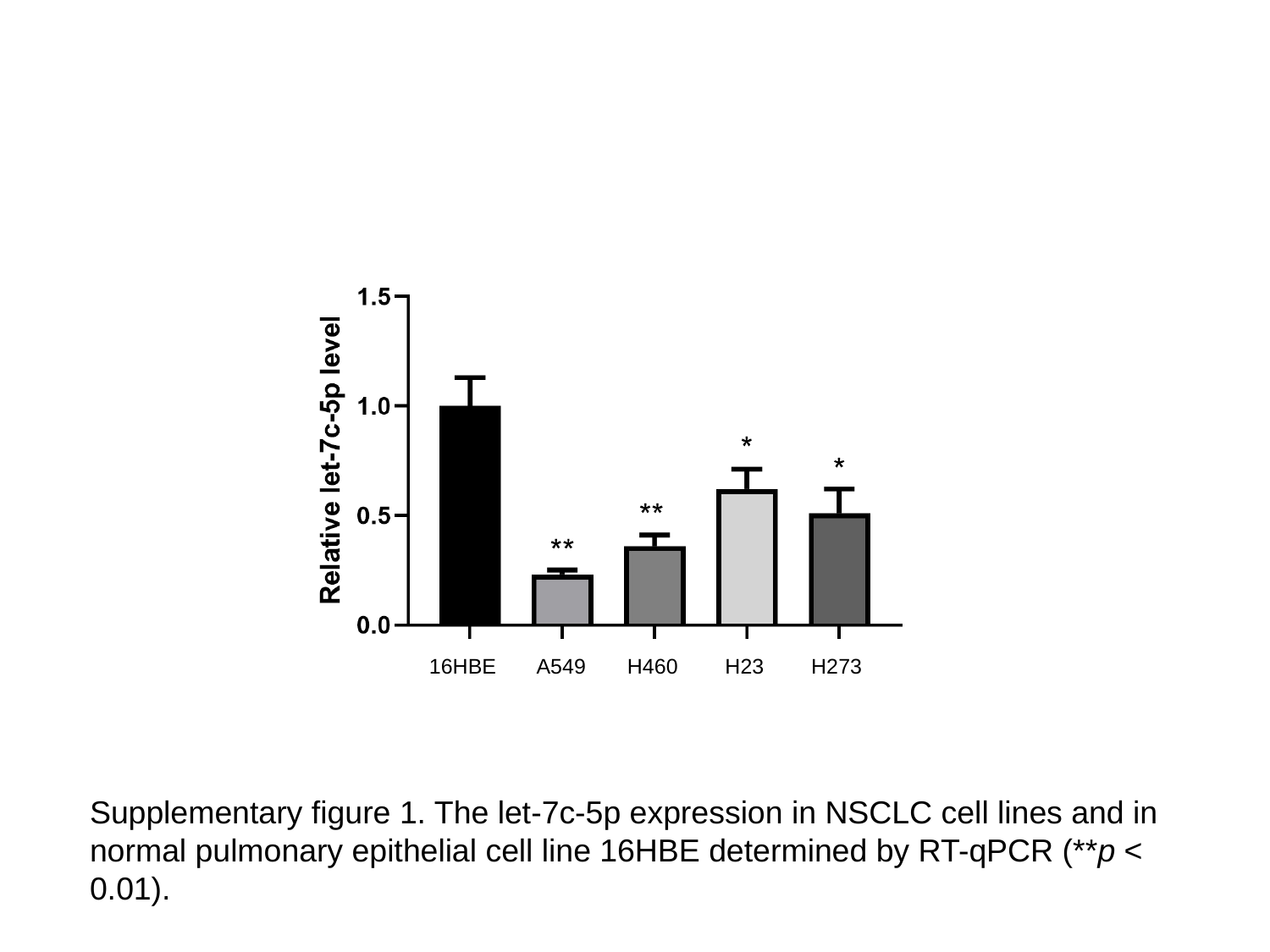

16HBE A549 H460 H23 H273
Supplementary figure 1. The let-7c-5p expression in NSCLC cell lines and in normal pulmonary epithelial cell line 16HBE determined by RT-qPCR (**p < 0.01).
