# Supplementary material for: Deciphering the let-7c-5p/RRM2 axis in lung adenocarcinoma: expression, prognosis, and immune landscape implications
Source: Front Oncol. 2025 Nov 20;15:1628429. doi: 10.3389/fonc.2025.1628429 (PMC12675248; doi:10.3389/fonc.2025.1628429)
Supplement: Supplementary file 3 [file Table1.doc]

Supplementary Table 1.

| **Reagents** | **Primer sequence（5‘-3‘）** |
| --- | --- |
| let-7c-5p | 5’-GAG GTA GTA GGT TGT ATG -3’  5’-GAA CAT GTC TGC GTA TCT C-3’ |
| RRM2 | 5’-GTGGAGCGATTTAGCCAAGAA-3’  5’-CACAAGGCATCGTTTCAATGG-3’ |
| GAPDH | 5’-TGT GTC CGT CGT GGA TCT GA-3’  5’-CCT GCT TCA CCA CCT TCT TGA-3’ |
