# Supplementary material for: Deciphering the let-7c-5p/RRM2 axis in lung adenocarcinoma: expression, prognosis, and immune landscape implications
Source: Front Oncol. 2025 Nov 20;15:1628429. doi: 10.3389/fonc.2025.1628429 (PMC12675248; doi:10.3389/fonc.2025.1628429)
Supplement: Supplementary file 4 [file Table2.doc]

| **Reagents** | **Vendor** | **Information** |
| --- | --- | --- |
| SiRNA-RRM2 | jtsbio Biotechnology | siRNA-NC  Sence:UUCUCCGAACGUGUCACGUTT  Anti-Sence:ACGUGACACGUUCGGAGAATT  siRNA-RRM2-1  Sence: GGCUCAGCUUGGUCGACAATT  Anti-Sence: UUGUCGACCAAGCUGAGCCTT  siRNA-RRM2-2  Sence: GGGAUGAAUUGCACUCUAATT  Anti-Sence: UUAGAGUGCAAUUCAUCCCTT |
| hsa-let-7c-5p mimics | jtsbio Biotechnology | UGAGGUAGUAGGUUGUAUGGUUCCAUAC  AACCUACUACCUCAUU |

Supplementary Table 2
